# Hydrogen Sulfide: Biogenesis, Physiology, and Pathology

**DOI:** 10.1155/2016/6549625

**Published:** 2016-04-11

**Authors:** Jin-Song Bian, Kenneth R. Olson, Yi-Chun Zhu

**Affiliations:** ^1^Department of Pharmacology, Yong Loo Lin School of Medicine, National University of Singapore, Singapore 117597; ^2^Department of Physiology, University of Notre Dame, 1234 Notre Dame Avenue, South Bend, IN 46617, USA; ^3^Department of Physiology and Pathophysiology, Fudan University Shanghai Medical College, 138 Yi Xue Yuan Road, Shanghai 200032, China

The seminal studies by Kimura's group in the late 1990s [1, 2] ushered in a new era of biological signaling mediated by hydrogen sulfide (H_2_S). H_2_S has been described as the third “gasotransmitter” along with nitric oxide (NO) and carbon monoxide (CO; [3]). While none of these molecules actually signal as a gas, their hydrophilicity and lipophilicity enable them to readily traverse intracellular compartments where they serve in a variety of autocrine and paracrine signals capacities.

Of the three, however, H_2_S is arguably the most chemically reactive and biologically diverse. As a weak acid, H_2_S exists as the dissolved gas and hydrosulfide anion (HS^−^) in nearly equal proportions in the circumneutral intracellular milieu. H_2_S is readily oxidized to per- and polysulfides (H_2_S_*n*_) forming a highly reactive group of molecules (reactive sulfide species, RSS) that readily form reversible covalent bonds, coordinate with metal ligands, and participate in a variety of redox reactions [4, 5]. H_2_S was key in the origin of life [6] and it remains as the only inorganic substrate used by eukaryotic cells to generate ATP [7]. H_2_S mediates many physiological functions in various biological systems including cytoprotection, neuromodulation, ischemic responses, and oxygen sensing. Exogenous application of H_2_S may protect cell function against ischemic and oxidant injuries. Despite the fact that the multiple roles of H_2_S in biology have been intensely discussed over the last twenty years, many questions remain to be resolved. The eclectic nature of H_2_S is featured in this special issue as a collection of original articles and reviews that examine a variety of the physiological functions of H_2_S as well as its therapeutic attributes.

The role of H_2_S in oxidative stress has been one of the main focuses over the last two decades. The review by Xie et al. focuses on the new understanding and mechanisms of the antioxidant effects of H_2_S based on recent reports. As a poor reducing agent, H_2_S may react directly with and quench superoxide, peroxynitrite, and other ROS. However, the direct antioxidant effect may not be its main function. H_2_S can scavenge free radicals via activation of both nonenzymatic (e.g., glutathione and thioredoxin) and enzymatic (e.g., superoxide dismutase, catalase, and glutathione peroxidase) antioxidants. Apart from stimulation of cellular antioxidant defenses, H_2_S may also inhibit the overproduction of ROS via modification of the structure of p66Shc at cysteine-59. This subsequently inhibits mitochondrial ROS generation in the mitochondria. Thus, this review provides new insights to better understand the important role of the H_2_S in oxidative stress and the related diseases.

Being gaseous molecules and mediators, both H_2_S and NO play important roles in various biological systems. While the individual signaling mechanisms mediated by H_2_S and NO in mammals are extensively studied, our understanding about the potential relationship between these two gasotransmitters is woefully incomplete. Nagpure and Bian reviewed recent research progress in the interactions between these two gaseous mediators. H_2_S may reduce oxidized NO leading to the formation of HSNO as an intermediate. Further reduction and direct displacement of HSNO by H_2_S result in the formation of another intermediate product, nitroxyl (HNO). Interestingly, HNO produces chemical and physiological functions different from NO and H_2_S. The role of this interaction in heart and vascular physiology and pathology is also summarized in this review.

In the original article by D. Wu et al., the authors studied the interaction between H_2_S and NO in vasculature using a novel H_2_S and NO conjugated donor, ZYZ-803. The authors found that, by releasing H_2_S and NO, ZYZ-803 time- and dose-dependently relaxed rat aortic rings. Mechanistic studies revealed that the effect of ZYZ-803 was mediated by cGMP. Interestingly, suppression of either H_2_S or NO generation with their respective inhibitors abolished the vasorelaxant effect of ZYZ-803. These interesting data suggest that H_2_S and NO generated from ZYZ-803 can cooperatively regulate vascular tone.

In the original article by P. Huang et al., the authors investigated whether H_2_S can prevent high-salt diet-induced renal injury. Dahl rats on a high-salt diet for 8 weeks developed hypertension and kidney injury with excessive oxidative stress and an obvious reduction of endogenous H_2_S. Exogenous application of H_2_S, however, decreased blood pressure and improved renal function. The authors believe that the beneficial effects of H_2_S resulted from Keap1/Nrf2 disassociation.

Sulfur dioxide (SO_2_) has also long been thought to be a toxic gas. A group of scientists in China recently reported that this gas can be produced endogenously. In the review article by Y. Huang et al., the authors summarized the physiological and pathological functions of SO_2_ in the cardiovascular system. These include regulation of vascular tone and heart contractility and its effects on disease status like systemic and pulmonary hypertension, atherosclerosis, and ischemic diseases. Based on these effects, they propose that endogenous SO_2_ may be a potential therapeutic target for cardiovascular diseases.

In the original article by N. Li et al., the authors examined the effect of H_2_S on protein expression of various H_2_S generating enzymes in both ischemic heart tissue and cultured cardiomyocytes during hypoxia. They found that NaHS treatment upregulated protein expression of various enzymes with different patterns in the two pathological situations. The authors believe that CSE may serve as the most important enzyme to regulate H_2_S production in myocardium. This is because when CSE was deleted, the compensatory expressions of CBS and 3-MST were not enough to rescue H_2_S levels and protect the heart. These interesting data suggest that exogenous H_2_S may alter the production of endogenous H_2_S and therefore protect the heart with a new mechanism.

Respiratory and gastrointestinal epithelia are directly exposed to environmental- or bacterial-derived H_2_S which can raise the H_2_S concentration in these tissues. E. Pouokam and M. Althaus discussed in their review article how epithelial cells quickly process and maintain H_2_S level to prevent excessive increases. They proposed a new concept that electrolyte and liquid transport machinery inside epithelia may serve as a fortress against potentially harmful higher level H_2_S. In this interesting review article, reactions to exposed H_2_S and potential physiological and pathophysiological implications were also discussed.

Vascular calcification (VC) is a refractory component of many cardiovascular diseases and is associated with hypertension (HT) and endoplasmic reticulum stress (ERS). In this original research article, R. Yang et al. induced VC and HT in rats with vitamin D_3_ and observed that daily i.p. injection of H_2_S (as NaSH) over 28 days prevented VC, HT, and conversion of vascular smooth muscle cells from a contractile to an osteoblast-like phenotype common in VC. H_2_S also prevented VC-induced downregulation of cystathionine *γ*-lyase and the authors presented evidence that suggested H_2_S also ameliorated ERS. This article adds yet another piece to the ever growing body of evidence that H_2_S is a general cardiovascular protectant.

Unfortunately, this issue can only touch on a few select aspects of H_2_S biology. However, we hope this broad-spectrum, albeit limited, approach serves to illustrate the myriad of physiological processes potentially influenced by H_2_S and entice new investigators into this exciting field.

*Jin-Song Bian* *Jin-Song Bian* *Kenneth R. Olson* *Kenneth R. Olson* *Yi-Chun Zhu* *Yi-Chun Zhu*
